# POEMS syndrome with multiple ganglioside/paraneoplastic antibodies misdiagnosed as CIDP: a case report

**DOI:** 10.3389/fonc.2026.1773709

**Published:** 2026-04-30

**Authors:** Mengmeng Fan, Jiaxiu He, Shihui Sun, Linyuan He, Zhao Zhong

**Affiliations:** Department of Neurology, Tangdu Hospital, The Fourth Military University, Xi’an, Shaanxi, China

**Keywords:** castleman disease, chronic inflammatory demyelinating polyneuropathy, immunofixation electrophoresis, monoclonal gammopathy, paraneoplastic neurological syndrome, POEMS syndrome, vascular endothelial growth factor

## Abstract

POEMS syndrome is a rare multisystem disorder characterized by polyneuropathy, organomegaly, endocrinopathy, monoclonal protein (M-protein) secretion, and skin changes. Its pathogenesis is driven by plasma cell dyscrasias and the dysregulation of vascular endothelial growth factor (VEGF). In contrast, chronic inflammatory demyelinating polyneuropathy (CIDP) is an immune-mediated condition that primarily affects peripheral nerve myelin, lacking the systemic involvement and hematological markers characteristic of POEMS syndrome. Given their overlapping neuromuscular manifestations—primarily progressive sensory loss and motor weakness—POEMS is frequently misdiagnosed as CIDP during initial presentation. This report presents a case of POEMS syndrome initially presenting with lower limb paresthesia. Through evaluation of the clinical course, specialized laboratory findings, and histopathological analysis, the diagnosis was clarified. By integrating this case with a comprehensive literature review, our study aims to enhance clinical vigilance and diagnostic accuracy in distinguishing POEMS syndrome from CIDP, two disorders with superficially similar neuropathic features but fundamentally different underlying etiologies.

## 1 Case presentation and clinical findings

### 1.1 History of present illness

A 57-year-old male patient was admitted to our hospital on 21 November 2024, with a 7-month history of numbness in both lower limbs. The symptoms began 7 months earlier with numbness of the right thumb and second toe, without a distinct cause, and gradually progressed to involve both feet. He reported no dizziness, headache, seizures, limb muscle pain, or motor deficits. The patient was admitted to the Shaanxi Provincial People’s Hospital, where electromyography revealed peripheral neuropathy of both lower limbs, with impaired motor and sensory function. Subsequently, the symptoms persisted, with numbness gradually progressing to 10 cm proximal to the bilateral malleoli. This was accompanied by soreness, swelling of the lower limbs, gait instability, and bilateral hand numbness. Occasionally, the patient went to bed wearing shoes; however, the patient denied limb myalgia or motor weakness. He subsequently presented to our hospital for treatment. He reported a weight loss of approximately 25 kg over the past year. He had no history of diabetes, coronary heart disease, hypertension, or other chronic illnesses.

### 1.2 Prehospital examination

*Electromyography*: peripheral neuropathy of the extremities (sensory > motor), demyelinating lesions with axonal damage, bilaterally symmetrical, lower limbs > upper limbs, distal predominance; abnormal F waves; abnormal sensory evoked potentials in all four limbs.

*Dynamic electrocardiogram*: mean heart rate of 101 beats/min; sinus tachycardia observed.

### 1.3 Physical and neurological examination (on admission)

*Vital signs*: normal temperature, heart rate, respiratory rate, and blood pressure.

*General examination*: cardiopulmonary and abdominal examinations were unremarkable. A palpable enlarged lymph node (1.5 × 2 cm) was detected in the right axilla.

*Neurological examination*:

- Mental status: alert, higher cortical functions intact.
- Cranial nerves: pupils equal and round bilaterally; direct and consensual light reflexes intact; accommodation-convergence reflex normal; and extraocular movements full, with horizontal nystagmus in both eyes on left gaze.
- Motor: upper limb strength grade 5/5; proximal lower limb strength grade 4/5; distal lower limb strength grade 5-/5; dorsiflexion strength grade 3/5; plantar flexion grade 4/5; knee extensors drade 5-/5; and knee flexors grade 3/5.
- Coordination: finger–nose and heel–knee–tibia tests were stable. Romberg test was positive.
- Sensory: hyperalgesia to pinprick below the knees; decreased vibration sensation below the knees. Biceps, triceps, brachioradialis, patellar, and Achilles tendon reflexes were absent. Palmomental reflex was positive bilaterally. Babinski’s sign was absent bilaterally.

### 1.4 Laboratory findings (post-admission)

*Routine screening*: complete blood count (platelets: 243 × 10^9^/L), liver and renal function, electrolytes, fasting blood glucose, and coagulation studies―all within normal limits.

*Specialized diagnostic panels*:

- Tumor markers: CA72-4, CA19-9, CA125, CEA, AFP, ferritin, NSE, CK19, and PSA―all within normal limits.
- Autoantibodies: antinuclear antibody panel (anti-dsDNA, anti-Sm, anti-SSA, anti-Scl70, anti-RO52, anti-PM, and anti-PCNA)―all negative.
- Vasculitis series: normal.

*Thyroid function*: elevated thyroid-stimulating hormone (TSH): 5.67 μIU/mL; thyroid microsomal antibody: 89.30 IU/mL; and thyroglobulin antibody: 324.00 IU/mL. Sex hormones were normal.

*Immunology*: serum immunofixation electrophoresis identified a monoclonal IgA-λ band. Serum protein electrophoresis: α2 globulin 10.2%, albumin 53.6%, α1 globulin 3.8%, β-globulin 9.3%, γ-globulin 23.1%, A/G ratio 1.16, and M-protein 0.0%.

*Cerebrospinal fluid (CSF) examination*:

- Routine: clear, colorless; globulin qualitative test was positive; WBC 10.0 × 10^6^/L; and total cells 2.1 × 10^8^/L.
- Biochemistry: chloride 130.4 mmol/L, glucose 3.45 mmol/L, lactate dehydrogenase 18 U/L, protein 1,708.3 mg/L, and adenosine deaminase 1 U/L.
- Immunology: IgG 245 mg/L and albumin 1,220 mg/L.
- Cytology: slightly increased lymphocytes/eosinophils and increased neutrophils (possible blood contamination).

### 1.5 Imaging studies

*Chest CT*: irregular soft tissue density noted in the anterior mediastinum; exudative changes in the left lung; consolidation in the left lower lobe; left-sided pleural effusion; multiple enlarged mediastinal lymph nodes; enlarged cardiac silhouette; pericardial effusion; and hepatosplenomegaly.

*PET/CT*: hypermetabolic lymphadenopathy involving multiple enlarged mediastinal stations (2–5) and hilar regions (station 10); splenomegaly; findings suggestive of lymphoma; bilateral emphysema, left upper lobe interstitial pneumonia, segmental atelectasis in the left lower lobe, bilateral dorsal atelectasis, bilateral pleural effusion, pericardial effusion, anemia; incidental findings (a renal cyst of the right kidney, prostate calcification, and spinal degeneration). No other sites of abnormal metabolic activity were identified within the mediastinal structures or the brain.

### 1.6 Antibody detection

*Peripheral neuropathy-related antibodies*: Anti-GD1b, GD2, GD3, GT1a, GT1b, and GQ1b IgM—all positive (1:200).

**Figure 1:**
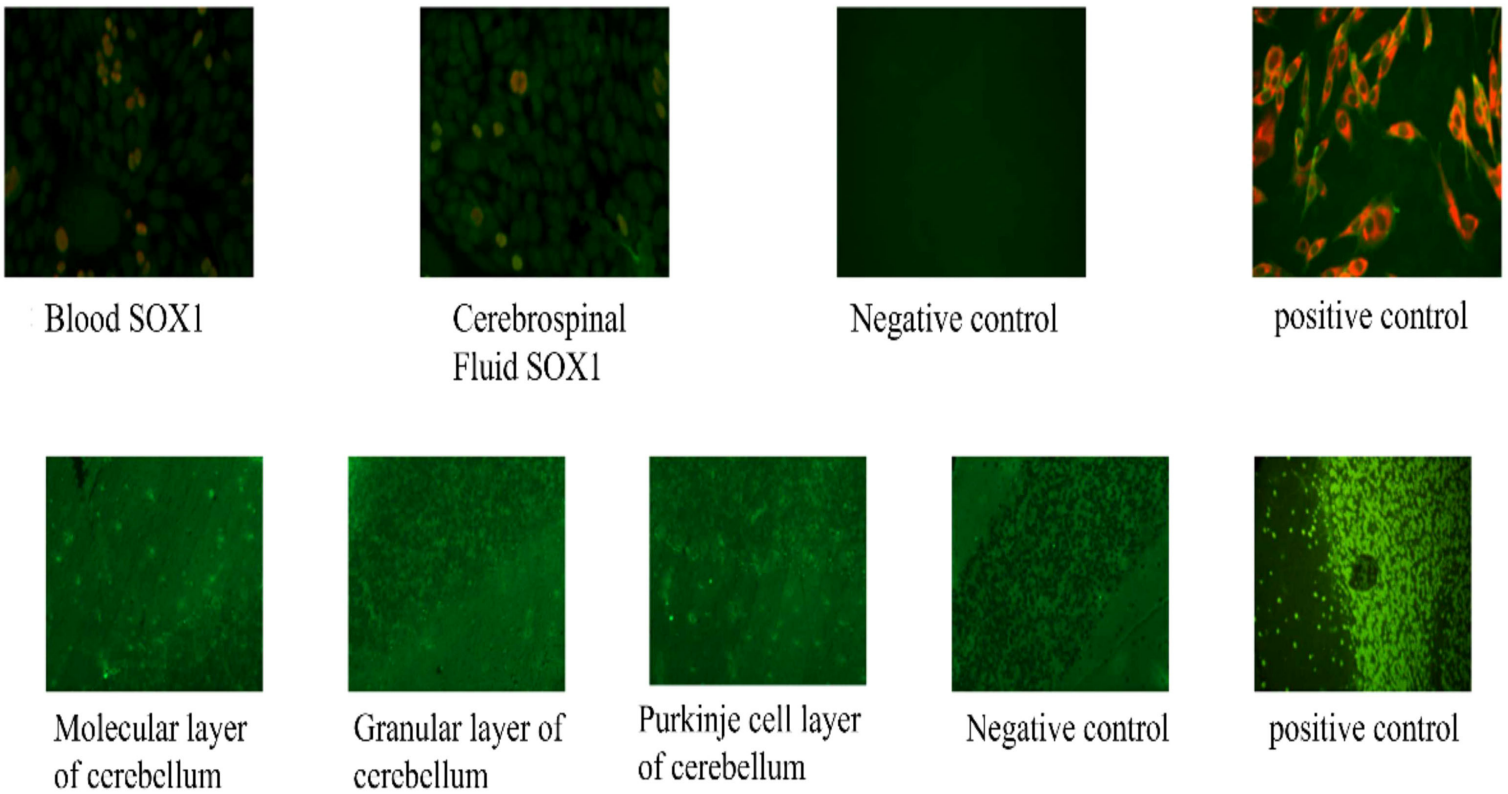
Peripheral neuropathy-related and paraneoplastic antibody detection. Top panel: representative immunofluorescence assay for anti-SOX1 antibodies: serum positive (1:100), cerebrospinal fluid weakly positive (1:1). Bottom panel: representative Tissue-Based Assay (TBA) for anti-neuronal antibodies: weakly positive (1:1) in the cerebellar molecular and granular layers.

*Paraneoplastic antibodies*: anti-SOX1 IgG positive in serum (1:100); weakly positive in CSF (1:1). Tissue-Based Assay (TBA): Anti-neuronal antibodies were weakly positive in the cerebellar molecular and granular layers (1:1 in both) shown in **Figure 1**.

## 2 Final diagnosis (Department of Neurology)

### 2.1 Peripheral neuropathy

2.1.1 Chronic inflammatory demyelinating polyneuropathy (CIDP).

2.1.2 Paraneoplastic peripheral neuropathy.

### 2.2 Lymphoma (probable)

## 3 Hospital course

During hospitalization, the patient received nerve nutrition therapy and hormone pulse therapy; despite treatment, limb numbness did not improve. The patient was referred to the Department of Hematology for further diagnostic evaluation.

### 3.1 Hematology evaluation

**Figure 2:**
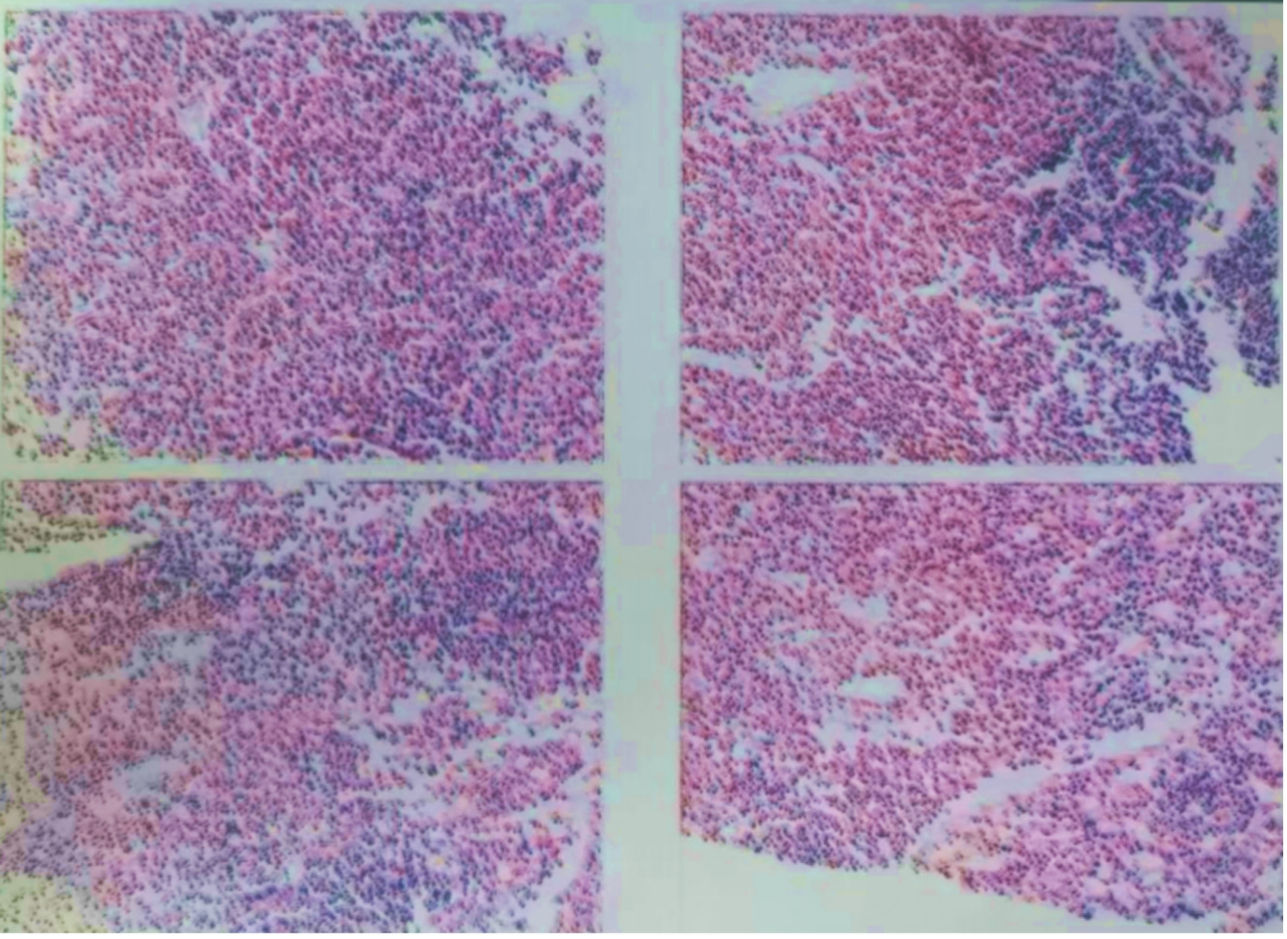
Pathological findings from the puncture tissue of the left anterior superior mediastinal mass, interpreted as giant lymph node hyperplasia (plasma cell type).

*Pathology*: needle biopsy of the left anterior superior mediastinal mass was performed. The pathological report indicated giant lymph node hyperplasia of the plasma cell type (**Figure 2**). Note: this was a small tissue puncture specimen with inherent limitations that may not represent the overall lesion.

*Immunohistochemistry:*

- CK19 (-), EMA (+, some cells), TdT (-), Ki-67 (+, ~8% of localized cells), and Vimentin (+).
- Lymphoid lineage markers: CD3 (+, some cells), CD5 (+, some cells), CD20 (+, some cells), and CD21 (+, FDC net).
- Germinal center/plasma cell markers: CD10 (-), Bcl-2 (+), Bcl-6 (+, few cells), and CD138 (+, some cells).
- Light chain restriction and NK/plasma cell markers: Kappa (+), Lambda (+, some cells), and CD56 (+, few cells).

*EBER in situ hybridization*: negative.

*Gene rearrangement*: suspicious clonal rearrangement of the immunoglobulin gene was detected; no clonal rearrangement of the TCR gene was identified.

Considered diagnosis: POEMS syndrome was considered based on the constellation of findings.

## 4 Treatment and outcome

The patient received two cycles of combined therapy with an anti-CD20 monoclonal antibody and a glucocorticoid. No significant improvement was observed in laboratory parameters or clinical symptoms, indicating ineffective treatment. The patient subsequently sought further medical care in Beijing, China.

## 5 Discussion

A 51-year-old man was admitted to our hospital with numbness and weakness in the lower extremities. Physical examination revealed a slight reduction in muscle strength in the bilateral feet and knees. Sensory evaluation demonstrated hyperesthesia below the knees, whereas vibratory sensation (as assessed by tuning fork) was diminished in the same distribution. Neurological signs included a positive Romberg sign (indicating sensory ataxia) and absent deep tendon reflexes (generalized areflexia).

Diagnostic workup was notable for the following:

- Electromyography: evidence of demyelinating disease and axonal injury.
- Imaging (CT and PET-CT): diffuse lymphadenopathy involving the mediastinal and hilar compartments, alongside bilateral pleural effusion and splenomegaly.
- Laboratory: autoimmune thyroid dysfunction and an IgA-λ monoclonal protein (M-protein) band on serum immunofixation.

While awaiting the lymph node pathology results, the patient was transferred from the Neurology Department to the Hematology Department under a differential diagnosis of peripheral neuropathy with suspected lymphoma. Histopathological analysis of the lymph nodes subsequently demonstrated a polymorphic lymphoplasmacytic infiltrate with polytypic (kappa and lambda) light-chain expression and a proliferation of follicular dendritic cells.

These findings, notably being HHV-8 negative, were consistent with idiopathic multicentric Castleman disease (iMCD), plasma cell type. When integrated with the patient’s multisystem clinical symptoms and auxiliary examinations, a final diagnosis of POEMS syndrome was established.

The peripheral neuropathy observed in iMCD typically manifests as a mild, length-dependent sensory polyneuropathy. Such subclinical presentations often lead to underdiagnosis; characteristic features include distal paresthesia and mild weakness, generally in the absence of neuropathic pain or significant motor involvement. In these cases, nerve conduction studies may reveal a primary demyelinating process (2). In contrast, POEMS syndrome-associated Castleman disease (CD) presents with a more severe, progressive sensorimotor polyneuropathy. Affected individuals may develop debilitating neuropathic pain, profound sensory deficits, and marked motor impairment. Electrophysiological studies in POEMS syndrome typically demonstrate primary demyelination with secondary axonal degeneration. In this case, the diagnostic criteria were met through the following triad: (I) mandatory chronic inflammatory demyelinating polyneuropathy, (II) MCD (plasma cell variant), and (III) a monoclonal plasma cell proliferative disorder (IgA-λ type; serum M-protein 1.2 g/dL). These findings fulfill the major diagnostic criteria for POEMS syndrome.

### 5.1 POEMS syndrome

POEMS syndrome is a rare multisystem disorder characterized by polyneuropathy, organomegaly, endocrinopathy, monoclonal protein, and skin changes. The condition, also known as Crow-Fukase syndrome, is fundamentally associated with an underlying plasma cell dyscrasia. It was initially described by Crow (6) in 1956 and later characterized in a landmark series of 102 cases by Nakanishi (3). The acronym POEMS syndrome was subsequently coined by Bardwick et al. in 1980 based on these defining clinical features (4).

According to the updated 2019 Mayo Clinic diagnostic criteria for POEMS syndrome (4), a definitive diagnosis requires the presence of both mandatory major criteria, at least one other major criterion, and at least one minor criterion.

- Mandatory major criteria: (I) peripheral polyneuropathy (demyelinating or axonal; typically CIDP); (II) monoclonal plasmacytic proliferative disorder (almost exclusively λ light-chain type).
- Other major criteria: (I) Castleman disease; (II) sclerotic bone lesions; (III) elevated vascular endothelial growth factor (VEGF).
- Minor criteria: (I) organomegaly (splenomegaly, hepatomegaly, or lymphadenopathy); (II) extravascular fluid overload (peripheral edema, pleural effusion, or ascites); (III) endocrinopathy (adrenal, thyroid, pituitary, gonadal, parathyroid, or pancreatic involvement); (IV) skin changes (hyperpigmentation, hypertrichosis, glomeruloid hemangiomas, plethora, acrocyanosis, or white nails); (V) papilledema; and (VI) thrombocytosis or polycythemia (erythrocytosis).

The 2011 diagnostic update for POEMS syndrome (5) recognized a CD variant, which can present without evidence of a detectable monoclonal plasma cell proliferative disorder. Following a multidisciplinary consensus, the patient was diagnosed with POEMS syndrome based on the fulfillment of both mandatory criteria (polyneuropathy and an IgA- λ monoclonal plasma cell disorder), one additional major criterion (MCD, plasma cell variant), and three minor criteria (organomegaly, extravascular fluid overload, and endocrinopathy).

### 5.2 Serological immunoprofile and autoantibody analysis

A notable feature of this case was the presence of multiple positive autoantibodies. Testing for anti-ganglioside antibodies revealed a broad IgM profile, including positivity for anti-GD1b, anti-GD2, anti-GD3, anti-GT1a, anti-GT1b, and anti-Gq1b. Furthermore, paraneoplastic neurological syndrome (PNS) screening identified anti-SOX1 IgG in the peripheral blood. Analysis of the CSF confirmed the presence of anti-SOX1 IgG alongside anti-neuronal antibodies (specifically targeting the cerebellar molecular and granular layers via Tissue-Based Assay [TBA]).

### 5.3 The significance of polyautoimmunity in POEMS syndrome

The immunological profile in this case was remarkably complex, characterized by multiple anti-ganglioside IgM antibodies and anti-SOX1 IgG positivity. Ganglioside antibodies (such as anti-GM1, GD1a, and GQ1b) are established biomarkers for peripheral neuropathy. Their pathogenic mechanisms are traditionally associated with molecular mimicry of pathogens, such as *Campylobacter jejuni*, leading to complement-mediated axonal or myelin damage (7, 8). In clinical practice, anti-GM1 antibodies are highly correlated with acute motor axonal neuropathy (AMAN) (9), while anti-GQ1b antibodies are highly specific for Miller–Fisher syndrome (a variant of Guillain–Barré syndrome) (10).

In the present case, the broad spectrum of positive IgM ganglioside antibodies (GD1b, GD2, GD3, GT1a, GT1b, and GQ1b) suggests a complex paraneoplastic immune response rather than a post-infectious etiology. While these antibodies are usually detected via ELISA or cell-based assays to guide the selection of first-line therapies, such as intravenous immunoglobulin (IVIG) or plasma exchange, their presence here likely synergizes with elevated VEGF and IL-6 levels to exacerbate the characteristic demyelinating polyneuropathy of POEMS syndrome. Furthermore, the co-occurrence of anti-SOX1 and cerebellar TBA positivity in the CSF indicates an unusually extensive overlap between POEMS and classic PNS.

PNS antibodies arise from a cross-reactive immune response between tumor-expressed antigens and neural antigens and indicate a strong association with underlying malignancies (11). Specifically, the anti-SOX1 antibody (also known as antiglial nuclear antibody, AGNA) is a high-risk onconeural marker strongly associated with small-cell lung cancer and paraneoplastic cerebellar degeneration (12) In the present case, the detection of anti-SOX1 in both serum and CSF—in the absence of the psychiatric symptoms typically described in the literature (13)—is a rare finding in POEMS; as a high-risk paraneoplastic marker, its presence necessitates rigorous longitudinal oncological surveillance. In clinical practice, whole-body PET-CT scans and tumor markers, such as neuron-specific enolase (NSE) and cancer antigen 125 (CA-125), should be utilized to screen for primary tumors. Treatment typically involves surgical resection of the primary tumor combined with immunotherapy regimens, such as rituximab and cyclophosphamide. Furthermore, regular monitoring of antibody titers may provide valuable insights into the risk of tumor recurrence or the emergence of an occult malignancy.

#### 5.3.1 The antibody─POEMS relationship: pathogenic interplay between monoclonal proteins and autoimmunity

A defining characteristic of POEMS syndrome is the presence of a monoclonal (M) protein, typically of the λ light-chain type. In the present case, initial serum protein electrophoresis (SPEP) revealed a polyclonal increase in the γ-globulin fraction (23.1%) without a quantifiable M-protein spike (0.0%). However, the more sensitive serum immunofixation electrophoresis (IFE) successfully identified a monoclonal IgA-λ band. This discrepancy is characteristic of POEMS syndrome, particularly the CD variant, where systemic manifestations are driven by a small, often non-quantifiable plasma cell clone. Notably, emerging evidence suggests that the M-protein may drive the production of autoantibodies through direct and indirect mechanisms, further complicating the clinical overlap with traditional autoimmune neuropathies such as CIDP.

Direct cross-reactivity and molecular mimicry: The M-protein secreted in POEMS syndrome (mostly λ light-chain type IgG or IgA) may directly target neural ganglioside epitopes through molecular mimicry within the antigen-binding variable region (Fab segment) of the immunoglobulin (14). If the Fab segment shares structural homology with glycosyl epitopes of gangliosides, it can simulate autoantibody binding, activate the complement system, and induce axonal or myelin damage (15, 16). Furthermore, if the Fab segment overlaps with epitopes of neuronal nuclear antigens (e.g., SOX1, Hu) or Purkinje cell antigens (e.g., Yo), it may induce cross-reactive autoantibodies that drive the observed paraneoplastic phenotype. While low-titer serum anti-Hu antibodies are reported in 5% to 10% of POEMS cases, in association with central nervous system involvement (17), the detection of anti-SOX1 in the present case highlights the unusually specific targeting of glial-associated transcription factors within the POEMS-MCD spectrum.

Vascular permeability and antigen exposure: VEGF serves as a central pathological driver in POEMS syndrome. Pathologically elevated VEGF levels can significantly increase vascular permeability and facilitate the infiltration of inflammatory cells and autoantibodies into the endoneurium (18, 19). This vascular “leakage” may cause the shedding of gangliosides from the axonal membrane into the systemic circulation, effectively breaking immune tolerance and stimulating the production of polyclonal anti-ganglioside antibodies (20). Moreover, the disruption of the blood-nerve and blood-brain barriers allows paraneoplastic antibodies (such as anti-SOX1 or anti-Ri) to enter the CSF and directly access central neurons or glial cells (20).

Immune microenvironment dysregulation and epitope spreading: Chronic inflammatory states and immune microenvironment dysregulation constitute additional contributors to antibody positivity. Clonal plasma cell proliferation is frequently accompanied by the excessive secretion of proinflammatory cytokines (e.g., IL-6, TNF-α), driving hyperactivation of B lymphocytes and expansion of autoreactive T-cell populations. Sustained inflammation mediated by M-protein may unveil cryptic ganglioside antigenic epitopes through epitope spreading mechanisms. This process effectively recruits and activates latent autoreactive B-cell clones, further diversifying the autoantibody profile (21).

### 5.4 Differential diagnosis: distinguishing POEMS syndrome from CIDP

Several key markers facilitate this differential diagnosis: the presence of an M-protein, elevated VEGF levels, organomegaly, endocrinopathy, and skin lesions—all hallmarks of POEMS that are characteristically absent in idiopathic CIDP. While CIDP is an immune-mediated demyelinating polyneuropathy affecting peripheral nerve myelin, it lacks the multisystem involvement and underlying plasma cell dyscrasia that define POEMS. A detailed comparison of these clinical features is provided in **Table 1**.

**Table 1:** Differential diagnostic criteria between CIDP and POEMS syndrome.

	CIDP	POEMS syndrome
Disease essence	Idiopathic, immune-mediated demyelinating neuropathy	Paraneoplastic multisystem syndrome secondary to a monoclonal plasma cell dyscrasia
Core diagnostic prerequisites	Polyradiculoneuropathy with objective evidence of demyelination (22)	Peripheral neuropathy AND monoclonal plasma cell disorder (mandatory) (23)
Onset pattern	Subacute or chronic; progressive or relapsing─remitting.	Chronic progressive; spontaneous remission not observed
Involvement pattern	Symmetric proximal and distal involvement; proximal weakness is often a hallmark	Distal-to-proximal involvement; distal involvement is more severe, with lower extremities affected significantly more than upper extremities
Sensory symptoms and pain	Predominantly large-fiber loss (numbness, tingling, ataxia); significant pain is rare	Small and large-fiber involvement; severe neuropathic pain and hyperalgesia (burning pain) are characteristic
Cranial nerve involvement	May involve the facial nerve, oculomotor nerve, or other cranial nerves	Rarely involves the cranial nerves
Tendon reflexes	Early loss or hyporeflexia	Distal hyporeflexia, generalized loss in advanced stages
Multisystem involvement	Absent; typically presents as a restricted neurological disease	Inevitable multisystem involvement.
Organomegaly	Absent	Common; involves hepatomegaly, splenomegaly, or lymphadenopathy
Skin changes	Absent	Common; includes hyperpigmentation, hirsutism, skin thickening, or glomeruloid hemangiomas.
Edema/effusion	Absent	Common; includes peripheral edema, pleural effusion, ascites, or papilledema
Endocrine abnormalities	Absent	Common; includes hypothyroidism, hypogonadism, adrenal insufficiency, or diabetes mellitus
Bone lesions	Absent	Characterized by osteosclerotic lesions. (note: may be absent in the Castleman Disease variant)
Castleman Disease	Absent	May be present; specifically characterizes the CD variant of POEMS syndrome
Cerebrospinal fluid (CSF) profile	Classic albuminocytologic dissociation (highly elevated protein with normal white cell count)	Protein is often elevated, but opening pressure is frequently increased. Albuminocytologic dissociation is less specific/diagnostic than in CIDP
M-Protein	Usually absent; a small monoclonal peak (MGUS) may be present in ~20% of cases	Mandatory; detected in nearly 100% of cases, predominantly IgA-λ or IgG-λ
Serum VEGF	Normal or slightly elevated	Markedly elevated; typically 5–30 times the upper limit of normal
Electrophysiology	Typical demyelination: Significant slowing of conduction velocity, conduction block, temporal dispersion, and F-wave abnormalities	Mixed demyelinating and axonal features; more severe distal axonal loss; tibial/sural nerves often unrecordable
Nerve pathology	Perivascular inflammatory cell infiltration, “onion bulb” formation (repeated cycles of demyelination/remyelination)	Minimal inflammation; vascular proliferation, intimal thickening, and presence of uncompacted myelin lamellae
First-line treatment	Glucocorticoids, IVIG, or plasma exchange with good response	Poor or transient response to glucocorticoids/IVIG
Core treatment	Immunomodulation: glucocorticoids, IVIG, or plasma exchange	Clone-directed therapy: targeted anti-plasma cell therapy (e.g., lenalidomide, bortezomib) or autologous stem cell transplantation
Relapse pattern	Common relapsing-remitting course	Continuous progression, rare spontaneous remission
Prognosis	Generally favorable; functional maintenance achievable	Progressive neurological and systemic deterioration without treatment, survival affected

Peripheral neuropathy (PN) serves as the obligatory diagnostic cornerstone of POEMS syndrome, with an incidence approaching 100% and being the initial presenting symptom in ~60% of cases. Electrophysiologically, a mixed demyelinating–axonal pattern—characterized by severe distal axonal loss (often resulting in unrecordable tibial/sural nerves) and the absence of conduction block—distinguishes POEMS from the typical findings in CIDP. Notably, subclinical PN detected by nerve conduction studies enables early identification before overt symptoms appear, which is critical for improving outcomes in this multisystem syndrome.

Beyond diagnosis, PN severity correlates with overall disease burden and VEGF levels, guiding staging and prognosis. Serum VEGF represents a pivotal differential diagnostic indicator between POEMS syndrome and CIDP, as supported by data from the Latin American multicenter GELAMM study. The failure to prioritize these biomarkers remains a clinical limitation that exacerbates the misdiagnosis of POEMS syndrome as CIDP—particularly given their overlapping polyneuropathic presentations.

## 6 Conclusion

POEMS syndrome is a rare multisystem disorder that frequently mimics CIDP. In patients presenting with progressive limb numbness and weakness as the chief complaint, clinicians should exercise caution when interpreting positive antibody results or elevated CSF protein levels that may lead to diagnostic anchoring. A lack of response to standard immunomodulatory therapy necessitates comprehensive screening for monoclonal plasma cell dyscrasias and systemic markers to ensure a timely and accurate diagnosis.

## Data Availability

This article had no dataset. Requests to access the datasets should be directed to Jiaxiu He, sherilyhe1996@163.com.
